# Pylons ablaze: Examining the role of 5G COVID‐19 conspiracy beliefs and support for violence

**DOI:** 10.1111/bjso.12394

**Published:** 2020-06-21

**Authors:** Daniel Jolley, Jenny L. Paterson

**Affiliations:** ^1^ Northumbria University UK

**Keywords:** COVID‐19, conspiracy theories, violence, anger, paranoia

## Abstract

Amid increased acts of violence against telecommunication engineers and property, this pre‐registered study (*N* = 601 Britons) investigated the association between beliefs in 5G COVID‐19 conspiracy theories and the justification and willingness to use violence. Findings revealed that belief in 5G COVID‐19 conspiracy theories was positively correlated with state anger, which in turn, was associated with a greater justification of real‐life and hypothetical violence in response to an alleged link between 5G mobile technology and COVID‐19, alongside a greater intent to engage in similar behaviours in the future. Moreover, these associations were strongest for those highest in paranoia. Furthermore, we show that these patterns are not specific to 5G conspiratorial beliefs: General conspiracy mentality was positively associated with justification and willingness for general violence, an effect mediated by heightened state anger, especially for those most paranoid in the case of justification of violence. Such research provides novel evidence on why and when conspiracy beliefs may justify the use of violence.

## Background

During the COVID‐19 pandemic, telecommunication masts across Europe, North America, and Australasia have been damaged or destroyed in arson attacks, while engineers have been subjected to verbal and physical abuse (Ankel, 2020; Cerulus, 2020; Pasley, 2020). Such violence not only seems unwarranted, attacking essential workers along with vital infrastructure, particularly during a global pandemic, is extremely irresponsible and dangerous (e.g., Cowburn, 2020). Why, then, have some individuals resorted to these senseless attacks? According to police officials and media commentators, the perpetrators are likely to be motivated by the erroneous conspiratorial belief that electromagnetic waves transmitted by 5G technology have somehow caused COVID‐19 and so respond with violent actions to stop, what they see, as the origin of COVID‐19 (e.g., Waterson & Hern, 2020). By empirically testing this assumption, the current research provides a timely and important investigation into the associations between conspiracy beliefs and violence to explore *why* and *when* conspiracy beliefs may justify – and ignite – violence.

Conspiracy theories explain the ultimate causes of significant events as the secret actions of malevolent groups, who cover‐up information to suit their interests (e.g., Douglas, *et al*., 2017). These beliefs tend to emerge in times of crisis in society (van Prooijen & Douglas, 2017), where people are seeking to make sense of a chaotic world (e.g., Franks, et al., 2017 ). With its ensuing worldwide chaos, COVID‐19 typifies the most fertile ground for conspiracy theories to bloom (see Van Bavel *et al*., 2020). Disconcertingly, the consequences of such conspiracy theories are significant and wide‐ranging (e.g., increase in prejudice and everyday crime; see Jolley, *et al.,* 2020) and may be linked to violent intentions. Uscinski and Parent (2014), for example, found that people high in conspiracy thinking were more supportive of political violence, while Imhoff *et al*. (2020) found that when people took the perspective that society is governed by conspiracies, they were more supportive of violent extremism. To date, however, the mechanisms linking conspiracy beliefs and violent intent have yet to be explored.

One possible mechanism between conspiracy beliefs and violent intent is likely to be anger. Anger is usually elicited when individuals perceive an entity is intentionally threatening or inflicting harm to the self or ingroup (Giner‐Sorolla, & Russell, 2019). As conspiracy theories depict ‘conspirators’ as hostile actors who seek to cause such intentional harm (e.g., van Prooijen & Douglas 2017), it is likely that conspiracy beliefs will evoke anger. In support of this assertion, conspiracy narratives have been shown to promote hostility in individuals (e.g., Abalakina‐Paap, *et al*., 1999; Marchlewska, *et al*., 2019), a construct related to anger (Rubio‐Garay, *et al*., 2016). Importantly, as anger can inspire people to redress perceived wrongdoings or injustices (Carver & Harmon‐Jones, 2009; Fischer & Roseman, 2007), often by motivating people to act to confront, hit, or attack the anger‐evoking target (Berkowitz, 1993; Mackie, *et al*., 2000; Roseman, *et al*., 1994), this anger is likely to provoke violence (Coid *et al*., 2013a; Reagu, *et al*., 2013; Ullrich *et al*., 2014). It is plausible, therefore, that conspiracy beliefs may increase feelings of anger which, in turn, could be associated with the increased support of violence. That is, subscribing to the viewpoint that powerful hostile *others* are conspiring (e.g., about the link between 5G and COVID‐19) is likely to increase anger which, in turn, evokes violence towards a specific target (e.g., telecommunications masts and engineers).

While anger is a likely mediator between conspiracy beliefs and violent intent, it is clear that anger does not always provoke violence. Indeed, anger promotes a range of behaviours including non‐violent responses (Halperin, 2008) and even positive, pro‐social responses (Van Doorn, *et al*., 2014). So, key to understanding – and tackling – violent responses to conspiracy beliefs is uncovering *when* conspiratorially evoked anger is most likely to trigger violence. One pertinent factor could be paranoia. As a distinct but closely correlated construct of conspiracy beliefs, paranoia refers to the belief that a wide range of external agents harbours hostile intent towards them *personally* – as opposed to the conspiratorial belief that powerful organizations are harming society at large (Imhoff & Lamberty, 2018). Such self‐referential paranoia, along with anger, has been identified as a significant predictor of violence in forensic psychological research (e.g., Doyle & Dolan, 2006). Notably, when investigating the link between paranoia and violence in a clinical sample, Coid et al. (2013b) found that violence was a more likely outcome when individuals experienced paranoia *and* were angry, thus suggesting a moderating effect between the two variables (see also Ullrich *et al*., 2014). Extrapolating from this clinical sample, then, suggests that conspiratorially provoked anger is most likely to be associated with violence for those reporting more paranoia.

### The present research

**Figure 1:**
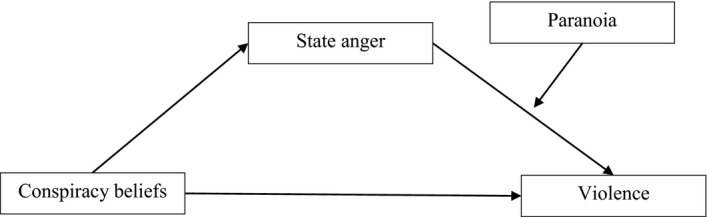
A path diagram to present the hypothesized moderated mediations with conspiracy beliefs (either 5G COVID‐19 conspiracy beliefs or conspiracy mentality) as predictors, state anger as mediator, and measures of justification and willingness for violence as criteria, with the *b*‐path moderated by paranoia.

Previous research has demonstrated that conspiracy theories may be linked with violent intentions (Uscinski & Parent, 2014). To date, however, we do not understand *why* conspiracy theorizing may be linked with violence, and *when* such a relationship may be more pronounced. The current research sought to address these gaps. Specifically, in a British sample, we hypothesize that 5G COVID‐19 conspiracy theory beliefs will be positively associated with the justification and willingness of real‐life violence, hypothetical violence, and the intention to be violent in response to the alleged link between 5G and COVID‐19 (*H1*), which will be mediated by higher levels of state anger (*H2*). We also posit that moderated‐mediational analyses will reveal that the associations between anger and violent responses within the mediational model will be strongest for those highest in self‐reported paranoia (*H3*). Finally, highlighting the generalizability of the research, we hypothesize that general conspiracy theorizing will be linked to general measures of violence, an effect explained by state anger (*H4*), which is similarly conditional on high levels of paranoia (*H5*). Figure 1 presents the hypothesized moderated mediations.

## Method

### Participants

All analyses were pre‐registered.^1^ The sample size was calculated using the linear multiple regression option in G*Power specifying three predictors and their product terms to examine conditional (moderated) effects at 80% power to find a small effect (.02), which is recommended to be a good first estimate in psychological research (Brysbaert, 2019). The recommended minimum sample was 550. We recruited 601 participants (436 females, 162 males, 2 trans, 1 non‐binary; *Mage* = 34.34, *SD* = 12.09; all UK residents) via the online participant database, *Prolific,* on 10 April 2020. Participants received a small participation fee. Education levels varied: 0.8% had no formal qualifications, 11% had GCSEs (or equivalent), 31.1% had A‐levels/BTEC, 40.4% had a degree, 13.8% had a Masters, and 2.8% had a PhD. One hundred and eighty‐three (30.4%) had, or knew someone who had, contracted COVID‐19.

### Materials and procedure

Unless otherwise stated, items were measured on a 1 (*strongly disagree*) to 7 (*strongly agree*) scale and were counterbalanced.^2^

General conspiracy mentality was measured using five items (e.g., ‘*I think that events which superficially seem to lack a connection are often the result of secret activities*’, α = .83, Bruder *et al*., 2013). Belief in 5G COVID‐19 conspiracy theories was measured with five items (e.g., ‘*The real truth about the link between COVID‐19 and 5G is being kept from the public*’, α = 97, adapted from Wood, 2017). Participants then completed the State Anger Scale (Spielberger & London, 1982), indicating how they felt *at the moment* using 15 items (e.g., ‘*I am mad*’, α = .96, 1 = *not at all*, 7 = *very much*).

Next, participants were presented with the following excerpt taken from the BBC but anonymized for the purpose of the study: ‘*Mobile phone mast fires are being investigated amid conspiracy theories claiming a link between 5G and coronavirus. There have been fires at masts in Birmingham, Liverpool and Melling in Merseyside*’. They were then asked ‘*Do you believe that the events described in the text are justified*’ (*1 = unjustified – 7 = justified;* justification of real‐life violence) and ‘*In the future, how likely is it that you would engage in behaviours described in the text*?’(*1 = very unlikely – 7 = very likely;* willingness for real‐life violence).

Afterwards, participants indicated whether they believed a range of behaviours were justified in response to the alleged link between 5G mobile technology and COVID‐19 using seven items (*1 = unjustified – 7 = justified)*. Cronbach’s alpha was unacceptable (α = .65) so an exploratory factor analysis (EFA) was conducted on all items. All statistical assumptions were met and two factors emerged (50.16% and 23.24% variance explained, respectively). On inspection, violent items were shown to be the first factor (five items: e.g., ‘*Arson attacks on private property*’; α = .87) and the second comprised of non‐violent items (two items: ‘*Boycotts of organisations you believe are responsible’; ‘Signing a petition to the authorities’,* Spearman–Brown coefficient = .86).

Participants were also asked how likely that they would engage in the same behaviours (*1 = very unlikely) – 7* = *very likely,* α = .67*)*. Again, an EFA was conducted which met statistical assumptions and two factors emerged (violent, α = .94 [62.03% of variance]; non‐violent, Spearman–Brown coefficient = .87 [23.31% of variance]). As the study’s hypotheses were formed for violent responses, the non‐violent items are reported in the supplementary materials.

Participants then completed two general measures of violence (Lamberty & Leiser, 2019): general justification of violence (3 items including, ‘*In certain situations, I am quite willing to use physical violence to assert my interests*’ α = .85) and general willingness to use violence (two items^3^ ‘*In general, I would be willing to use physical violence to fight others’; ‘I think it’s good if there are people who also use violence to bring back order’,* Spearman–Brown coefficient = .74). Finally, participants completed a measure of paranoia (Paranoid Ideation Scale, Fenigstein & Vanable, 1992) using twenty items (e.g., ‘*Someone has it in for me*’, α = .92), each on a 7‐point scale (*1 = not at all applicable to me, 7 = extremely applicable to me*). Demographic questions then followed.

## Results

### Data checking and correlations

As some variables exhibited significant skew, non‐parametric analyses were performed on the data. Table 1 presents the descriptives and Spearman’s rank correlations. In support of *H1*, belief in 5G COVID‐19 conspiracy theories was significantly and positively correlated with all measures assessing justification and willingness for violent responses to 5G COVID‐19 conspiracy theories. Likewise, conspiracy mentality was positively correlated with all measures. Supporting the proposed mediational patterns (*H2* and *H4*), state anger was positively associated with the proposed predictors (belief in 5G COVID‐19 conspiracy theories and conspiracy mentality), as well as all the proposed criteria (i.e., the justification and willingness for violent responses). Correlational analyses also revealed that participant demographics (age, gender, education level, and experience with COVID‐19) significantly correlated with a variety of measures and so were controlled for in the subsequent analyses.

**Table 1:** Descriptives statistics and Spearman’s rank correlations across variables

	1.	2.	3.	4.	5.	6.	7.	8.	9.	10.
1. Conspiracy mentality	–	.51*	.21*	.30*	.18*	.22*	.13*	.17*	.17*	.27*
2. Belief in 5G COVID‐19 CT		–	.16*	.53*	.31*	.30*	.18*	.03	−.04	.18*
3. State anger			–	.17*	.14*	.21*	.14*	.15*	.11*	.37*
4. Justification of real‐life violent responses to 5G COVID‐19 CT				–	.48*	.36*	.22*	.10*	−.01	.18*
5. Willingness of real‐life violent responses to 5G COVID‐19 CT					–	.30*	.25*	.16*	.09*	.13*
6. Justification of violent responses to 5G COVID‐19 CT						–	.37*	.27*	.17*	.25*
7. Willingness for violent responses to 5G COVID‐19 CT							–	.17*	.13*	.16*
8. Justification of general violence								–	.63*	.29*
9. Willingness for general violence									–	.26*
10. Paranoia										–
*M* (SD)	4.43 (1.16)	1.93 (1.38)	2.08 (1.20)	1.72 (1.22)	1.23 (0.74)	1.11 (0.41)	1.04 (0.35)	1.79 (1.11)	2.03 (1.28)	2.61 (1.03)

* *p* < .05.

### Mediation: 5G COVID‐19 conspiracy beliefs, state anger, and violence

We examined the proposed mediational role of state anger between 5G COVID‐19 conspiracy beliefs and violent responses to the alleged link between 5G mobile technology and COVID‐19 (*H2*). As PROCESS is robust to non‐parametric data and statistical outliers (Demming, Jahn, & Boztug, 2017), PROCESS model 4 with 95% bias‐corrected confidence intervals and 5000 bootstrap samples were used (Hayes, 2013). Table 2 reveals strong support for *H2* showing that state anger was a significant mediator between all the measured variables. In addition, Table 2 shows that even accounting for these significant mediational pathways, 5G COVID‐19 conspiracy beliefs remained significantly and directly associated with all the criteria.^4^

**Table 2:** Total, direct, and indirect effects of conspiracy beliefs predicting violent responses, mediated by anger

Predictor	Criterion	Total effect	Direct effect	Indirect effect
5G COVID‐19 CT	Justification of real‐life violent responses to 5G COVID‐19 CT	**.44 [.37, .52]**	**.43 [.36, .51]**	**.01 [.0001, .03]**
	Willingness for real‐life violent responses to 5G COVID‐19 CT	**.19 [.14, .24]**	**.18 [.13, .23]**	**.01 [.0005, .03]**
	Justification of violent responses to 5G COVID‐19 CT	**.09 [.06, .12]**	**.08 [.05, .11]**	**.01 [.0007, .02]**
	Willingness for violent responses to 5G COVID‐19 CT	**.07 [.04, .09]**	**.06 [.04, .08]**	**.01 [.0002, .02]**
Conspiracy mentality	Justification of general violence	**.19 [.12, .26]**	**.16 [.09, .23]**	**.03 [.008, .05]**
	Willingness for general violence	**.20 [.12, .28]**	**.18 [.10, .26]**	**.02 [.0004, .04]**

Note

Significant effects are bolded for ease of viewing. CT = conspiracy theory. 95% bias‐corrected confidence intervals used, along with 5000 bootstrap samples. Controlling for age, gender, education, and experience with COVID‐19. Conspiracy mentality also used as a covariate when 5G COVID‐19 conspiracy theory as predictor.

### Moderated mediation: 5G COVID‐19 conspiracy beliefs, state anger, violence, and paranoia

To examine the hypothesis that the pathways between anger and the justification and willingness for violent responses in the mediations (i.e., the *b‐*paths) would be strongest for those highest in self‐reported paranoia (*H3*), we used PROCESS model 14 with 95% bias‐corrected confidence intervals and 5000 bootstrap samples (Hayes, 2013). In support of *H3*, Table 3 shows significant indices of moderated mediations for the justification and willingness of both real‐life violence and a range of other violent acts in response to the alleged 5G COVID‐19 link (though willingness for violent responses was marginal = .004, 95% bias‐corrected confidence intervals = −.0001, .01). Examining the conditional indirect effects at the three levels of the moderator (*M* and *M* ‐/+ 1*SD*) shows that, as hypothesized, the association between anger and violence was strongest for those who reported being more paranoid (though there was a marginal link between anger and the justification of real‐life violence for relatively highly paranoid participants). Similar to the mediational analyses, 5G COVID‐19 conspiracy beliefs again remained a significant direct and positive predictor of all the criteria even when accounting for the moderation‐mediational associations.

**Table 3:** Conspiracy beliefs predicting violent responses, mediated by anger, with the b‐paths moderated by paranoia

Criterion	Predictor	Coefficient	Index of moderated mediation	Conditional indirect effects at levels of paranoia		
				Low	Moderate	High
Justification of real‐life violent responses to 5G COVID‐19 CT	5G COVID‐19 CT	**.43 [.36, .51]**				
	Anger	−.10 [−.30, .10]				
	Paranoia	−.01 [−.17, .15]				
	Anger × Paranoia	.05 [−.01, .11]	**.01 [.0000, .02]**	−.003 [−.02, .01]	.003 [−.005, .02]	***.010 [−.0002, .03]***
Willingness for real‐life violent responses to 5G COVID‐19 CT	5G COVID‐19 CT	**.18 [.13, .23]**				
	Anger	−.09 [−.22, .03]				
	Paranoia	−.09 [−.20, .01]				
	Anger × Paranoia	.**06 [.02, .10]**	**.007 [.0000, .04]**	−.001 [−.02, .01]	.006 [−.001, .02]	**.010 [.001, .04]**
Justification of violent responses to 5G COVID‐19 CT	5G COVID‐19 CT	.**08 [.05, .11]**				
	Anger	**−.10 [−.18, −.03]**				
	Paranoia	**−.09 [−.15, −.03]**				
	Anger × Paranoia	**−.004 [−.007, −.002]**	**.01 [.0003, .02]**	−.002 [−.01, .01]	**.005 [.000, .02]**	**.010 [.001, .03]**
Willingness for violent responses to 5G COVID‐19 CT	5G COVID‐19 CT	**.06 [.03, .08]**				
	Anger	−.06 [−.12, .005]				
	Paranoia	**−.06 [−.12, −.01]**				
	Anger × Paranoia	**.03 [.01, .05]**	. ***004 [−.0001, .01]***	−.001 [−.01, .01]	.003 [−.001, .02]	**.007 [.0003, .02]**
Justification of general violence	Conspiracy mentality	**.14 [.07, .22]**				
	Anger	−.14 [−.33, .05]				
	Paranoia	−.02 [−.17, .13]				
	Anger × Paranoia	.**07 [.01, .13]**	**.02 [.005, .04]**	−.007 [−.03, .02]	.01 [−.007, .03]	**.03 [.01, .06]**
Willingness for general violence	Conspiracy mentality	**.15 [.06, .23]**				
	Anger	−.12 [−.34, .10]				
	Paranoia	.10 [−.07, .28]				
	Anger × Paranoia	.05 [−.02, .12]	.01 [−.005, .03]	‐	‐	‐

Note

Significant effects are bolded, and marginal effects are bolded and italicized for ease of viewing. CT = conspiracy theory. 95% bias‐corrected confidence intervals used, along with 5000 bootstrap samples. Controlling for age, gender, education, and experience with COVID‐19. Conspiracy mentality also used as a covariate when 5G COVID‐19 conspiracy theory was a predictor. Levels of the moderator are *M*–1*SD* (low paranoia), *M* (moderate paranoia), and *M* + 1*SD* (high paranoia).

### General conspiracy theorizing, state anger, general violence, and paranoia

Similar to the specific 5G COVID‐19 conspiracy beliefs, the more general measure of conspiracy mentality was found to be associated with justification and willingness for general violence, mediated by anger, thus supporting *H4* (Table 2). In addition, providing partial support for *H*5, Table 3 showed a significant index of moderated mediation for the justification of general violence variable. Replicating the findings above, this moderated mediation revealed that those highest in paranoia showed the strongest link between anger and the justification of general violence. However, there was no moderated‐mediation evident for the willingness for general violence measure. Conspiracy mentality, meanwhile, remained a direct and positive predictor of justification and willingness of general violence.

## Discussion

Police officials and media commentators worldwide have strongly proposed a link between acts of arson on 5G telecommunication masts and belief in 5G COVID‐19 conspiracy beliefs (e.g., Waterson & Hern, 2020). Our findings provide empirical support for such an assertion: belief in 5G COVID‐19 conspiracy theories were positively associated with such violent responses, mediated by state anger, especially for individuals highest in paranoia. These patterns were also largely replicated when exploring the link between general conspiracy theorizing and general violence, thus highlighting the potential generalizability of these associations.

Our findings make several important advances on previous work. Notably, we expand upon previous literature (e.g., Uscinski & Parent, 2014) by investigating the possible violent consequences of subscribing to a *specific*, as well as a general, conspiracy theory (i.e., 5G COVID‐19 beliefs). Further, to the authors' knowledge, this is the first time that the affective mechanisms (e.g., anger) between conspiracy beliefs and violence have been explored. Thus, our work makes a timely contribution not only to understanding the link between conspiracy beliefs and real‐life violence, but it also highlights the often overlooked yet important potential interplay between conspiracy theorizing and emotion.

In addition to identifying *why* conspiracy theorizing may promote violence, our findings help identify *who* is most likely to perpetuate conspiracy‐related violence. Supporting previous research with a clinical sample (Coid *et al*., 2013b), we present relatively consistent evidence that individuals who are most paranoid are most likely to respond violently to conspiratorially evoked anger. These findings are notable because of their novelty and their possible practical implications. Drawing on the anger management literature (DiGiuseppe & Tafrate, 2003), for example, it is possible that future research could explore interventions that target and teach paranoid individuals to respond to the anger they feel in response to conspiracy beliefs in more appropriate ways, thus reducing the likelihood of violence. This may be a particularly promising first step in combatting violent reactions considering that conspiracy beliefs are resistant to change (Jolley & Douglas, 2017), and currently, little is known of the links between conspiracy beliefs and anger – or how to reduce it. Thus, targeting the link between anger and violence may be a more effective initial approach.

Future research could also address our limitations. First, experimental and longitudinal designs would help strengthen our cross‐sectional claims. The low means of some of the more extreme questions may also cause concern, however, statistical tests robust to issues surrounding such skewed, infrequent data, still found consistent, albeit small, effects, even after controlling for key demographic variables. While the statistical effects may be small, they are nonetheless important because the impact of conspiracy‐inspired violence could be far‐reaching and significant. For example, British government officials warned that recent arson attacks against phone masts disrupted emergency services, endangering lives (Devlin, 2020). Thus, understanding any significant contributing factor, no matter how small, is important. Relatedly, future research could use more specific operationalizations of the constructs to identify larger effects, for example, instead of measuring anger felt in the moment, a more specific measure of anger directed at a particular group could be more informative. Future research could also explore other mechanisms between conspiracy theorizing and violence – such as fear and anxiety (Hatfield & Dula, 2014; Roberton, *et al*., 2012). Furthermore, as conspiracy‐related violence has become more worldwide, our findings both in relation to general conspiracy mentality and specific 5G COVID‐19 beliefs suggest that future research could replicate the effects in other contexts and with different conspiracy beliefs.

In summary, we provide the first empirical evidence suggesting that belief in 5G COVID‐19 conspiracy theories is associated with violent responses to the alleged link between 5G mobile technology and COVID‐19. This relationship is explained by state anger, where the effect between anger and violence is strongest for those who have heightened paranoia. This pattern is replicated for the link between conspiracy mentality and the justification of violence in general. Our novel findings not only extend previous research by examining the impact of conspiracy beliefs and violence on a topical issue, they also uncover *why* (anger) and *when* (paranoia) conspiracy beliefs may justify the use of violence. By building upon these findings, future research is well placed to explore interventions to mitigate the relationships between conspiracy beliefs, anger, and violence.

## Conflict of interest

The authors confirm they have no conflict of interest to declare. Authors also confirm that this article adheres to ethical guidelines specified in the BPS Code of Ethics and Conduct as well as the authors institution's ethics guidelines.

## Authors’ contribution

Daniel Jolley (Conceptualization; Formal analysis; Investigation; Funding acquisition; Project administration; Writing – original draft; Writing – review & editing). Jenny L. Paterson (Conceptualization; Formal analysis; Writing – original draft; Writing – review & editing).

## Supporting information


**Appendix S1.** Supplementary Material.

## Data Availability

The data that support the findings of this study are openly available on the Center for Open Science: Open Science Framework at https://osf.io/9tn57

## Footnotes

^1^
Pre‐registration: https://aspredicted.org/blind.php?x=aq8qn7

^2^
The study also assessed vulnerable narcissism as a proposed moderator, and compliance to confinement as an outcome for pre‐registered secondary analyses which are presented in the supplementary materials.

^3^
The item ‘*I would never use physical violence myself’* was removed and improved scale reliability from α = .60 to the acceptable threshold.

^4^
This pattern was replicated (alongside the moderated mediations) with a measure of general conspiracy theorising (see Supplementary Materials).
