# Supplementary material for: Pylons ablaze: Examining the role of 5G COVID‐19 conspiracy beliefs and support for violence
Source: Br J Soc Psychol. 2020 Jun 21;59(3):628–40. doi: 10.1111/bjso.12394 (PMC7323354; doi:10.1111/bjso.12394)
Supplement: Supplementary file 1 — Appendix S1. Supplementary Material. [file BJSO-59-628-s001.docx]

**Supplementary analyses**

Pylons Ablaze:

Examining the role of 5G COVID-19 conspiracy beliefs and support for violence

**Contents**

[The rationale for additional measures and analyses 3](#_Toc41503557)

[References 6](#_Toc41503558)

[Table S1. Descriptives statistics and Spearman’s Rank correlations across variables. 7](#_Toc41503559)

[Table S2. Total, direct, and indirect effects of 5G COVID-19 conspiracy beliefs predicting non-violent responses, mediated by anger 8](#_Toc41503560)

[Table S3. Conspiracy beliefs predicting non-violent responses, mediated by anger, with the b-paths moderated by paranoia 9](#_Toc41503561)

[Table S4. Total, direct, and indirect effects of conspiracy mentality predicting violent and non-violent responses to an alleged link between 5G and COVID-19, mediated by anger 11](#_Toc41503562)

[Table S5. Conspiracy mentality predicting violent and non-violent responses to an alleged link between 5G and COVID-19, mediated by anger, with the b-paths moderated at three levels of paranoia 12](#_Toc41503563)

[Table S6. 5G COVID-19 conspiracy beliefs predicting violent and non-violent responses, mediated by anger, with the b-paths moderated at three levels of narcissism 15](#_Toc41503564)

[Table S7. Conspiracy mentality predicting violent and non-violent responses, mediated by anger, with the b-paths moderated at three levels of narcissism 18](#_Toc41503565)

[Table S8. Total, direct, and indirect effects of conspiracy beliefs predicting compliance to confinement instructions, mediated by anger 21](#_Toc41503566)

[Table S9. Conspiracy beliefs predicting compliance to confinement, mediated by anger, with the b-paths moderated at three levels of the moderator 22](#_Toc41503567)

## **The rationale for additional measures and analyses**

**COVID-19 conspiracy beliefs, non-violent behaviour, anger and paranoia**

As reported in the main text, whilst we anticipated a unidimensional violence measure^[[1]](#footnote-1)^, the Explortorary Factor Analysis uncovered there were two sub-scales – violent (as reported in the main text) and non-violent behaviour. As the non-violent responses were not part of our pre-registered hypotheses, we report them here. Correlations demonstrated that non-violent behaviours were associated with all measured variables (see Table S1), except for compliance with confinement (an exploratory variables discussed below), and in the case of willingness for non-violent behaviour, there was no association with a willingness for violent behaviour. Next, as shown in Table S2, state anger was a significant mediator for willingness for non-violent responses, but not justification. We also found that levels of paranoia did not moderate the relationships (Table S3). In sum, though not a focus of the current study, we found inconsistent mediational relationships for *non-violent* responses that future research could explore.

**Conspiracy mentality, violent (and non-violent) behaviour, anger and paranoia**

We pre-registered^1^ secondary analyses to examine associations between conspiracy mentality and the measured justification and willingness for violent responses to 5G COVID-19 conspiracy theories, alongside mediations and moderated-mediations. Correlations demonstrated that conspiracy mentality was associated with all measured variables (see Table S1). Following our predictions, as shown in Table S4, and replicating the effects shown with 5G COVID-19 conspiracy beliefs in the main text, state anger was a significant mediator for all measured variables, except justification of non-violent responses. We also found that heightened levels of paranoia moderated the relationship between anger and violence, in a test of moderated-mediation (see Table S5). This secondary analysis provides further confidence in our analyses reported in the main text with 5G COVID-19 conspiracy beliefs.

**Vulnerable narcissism**

We also pre-registered^1^ whether vulnerable narcissism - which is characterised by a vulnerable sense of self and insecurity, and proneness to experiencing increased levels of anger and hostility (e.g., Krizan & Johar, 2015) - acted as moderator of the effect between anger and violence. Vulnerable narcissism was measured with 16 items, including “*When people don’t notice me, I start to feel bad about myself*” on a 1 (*strongly disagree*) – 7 (*strongly agree*) scale (α = .93, Schoenleber et al., 2016). Vulnerable narcissism was positively correlated with all measures (except compliance with confinement; see Table S1). As shown in Table S6 and S7, the effects were inconsistent during the test of moderated-mediation; vulnerable narcissism only acted as a moderator between anger and the justification of violent responses to 5G COVID-19 conspiracy theories (with 5G COVID-19 conspiracy beliefs and conspiracy mentality as predictors, respectively). Unlike paranoia which acted as a moderator in each analysis, vulnerable narcissism does not seem to moderate the effects.

**Compliance with confinement**

Based on previous literature, people who subscribe to conspiracy beliefs are less likely to follow recommended advice (e.g., vaccination, Jolley & Douglas, 2014), and instead, would prefer to engage with non-normative behaviours (Imhoff et al., in press). We pre-registered^1^ the prediction that conspiracy beliefs would be negatively correlated with confinement; an effect mediated by state anger. We further theorised that the relationship between anger and confinement would be conditional on paranoia and vulnerable narcissism. This variable was measured using one item: *“To what extent are you complying with the confinement measures that are currently in place in the UK i.e., limiting movement, limiting social contact”* (1 = *Not at all* to 7 = *Very much*; Marinthe, et al., in press).

As shown in Table S1, compliance with confinement was negatively correlated with justification and willingness of real-life violence, and justification of violent responses to 5G COVID-19 conspiracy theories. Belief in 5G COVD-19 conspiracy beliefs was also marginally negatively correlated with confinement (*rho* = -.07, *p* = .059). State anger was shown not to mediate the effects between conspiracy beliefs and confinement (Table S8). We did find, however, that paranoia moderated the relationship between anger and confinement in a test of moderated-mediation: conspiracy theories (COVID-19 and general mentality) was correlated with an increase in state anger, which was associated with higher engagement with confinement, for those who were low in paranoia (Table S9).

Our analyses demonstrate that COVID-19 conspiracy beliefs are a marginal predictor of less engagement with confinement (although the direct effect in the moderated mediation was shown to be significant); however, the interplay between anger and paranoia can alleviate the negative impact of the conspiracy belief.

## References

Imhoff, R., Dieterle L., & Lamberty, P. (in press). Resolving the Puzzle of Conspiracy Worldview and Political Activism: Belief in Secret Plots Decreases Normative but Increases Nonnormative Political Engagement. *Social Psychological and Personality Science.* doi: doi.org/10.1177/1948550619896491

Jolley, D., & Douglas, K. M. (2014). The Effects of Anti-Vaccine Conspiracy Theories on Vaccination Intentions. *PLoS ONE, 9,* e89177. doi: 10.1371/journal.pone.0089177

Krizan, Z., & Johar, O. (2012). Envy divides the two faces of narcissism. Journal of Personality, 80, 1415-1451

Marinthe, G., Brown, G., Delouvée, S., & Jolley, D. (in press). Looking out for Myself: Exploring the Relationship Between Conspiracy Mentality, Perceived Personal Risk and COVID-19 Prevention Measures*. British Journal of Health Psychology.* https://doi.org/10.1111/bjhp.12449

Schoenleber, M., Roche, M. J., Wetzel, E., Pincus, A. L., & Roberts, B. W. (2015). Development of a brief version of the Pathological Narcissism Inventory. *Psychological Assessment, 27(*4), 1520–1526. https://doi.org/10.1037/pas0000158

# Table S1. Descriptives statistics and Spearman’s Rank correlations across variables.

|  | 1. | 2. | 3. | 4. | 5. | 6. | 7. | 8. | 9. | 10. | 11. | 12. | 13. | 14. |
| --- | --- | --- | --- | --- | --- | --- | --- | --- | --- | --- | --- | --- | --- | --- |
| 1. Conspiracy mentality | - | .51*** | .21*** | .30*** | .18*** | .22*** | .13** | .36*** | .39*** | .17*** | .17*** | .27*** | -.02 | .21*** |
| 2. Belief in 5G COVID-19 CT |  | - | .16*** | .53*** | .31*** | .30*** | .18*** | .34*** | .50*** | .03 | -.04 | .18*** | -.08^†^ | .11** |
| 3. State anger |  |  | - | .17*** | .14*** | .21*** | .14*** | .14*** | .21*** | .15*** | .11** | .37*** | -.03 | .38*** |
| 4. Justification of real-life violent responses to 5G COVID-19 CT |  |  |  | - | .48*** | .36*** | .22*** | .29*** | .40*** | .10* | -.01 | .18*** | -.08* | .09* |
| 5. Willingness of real-life violent responses to 5G COVID-19 CT |  |  |  |  | - | .30*** | .25*** | .10* | .22*** | .16*** | .09* | .13** | -.10* | .10* |
| 6. Justification of violent responses to 5G COVID-19 CT |  |  |  |  |  | - | .37*** | .26*** | .31*** | .27*** | .17*** | .25*** | -.12* | .20*** |
| 7. Willingness for violent responses to 5G COVID-19 CT |  |  |  |  |  |  | - | .15*** | .26*** | .17*** | .13** | .16*** | -.08 | .11** |
| 8. Justification of non-violent responses to 5G COVID-19 CT |  |  |  |  |  |  |  | - | .63*** | .15*** | .09* | .22*** | .03 | .16*** |
| 9. Willingness for non-violent responses to 5G COVID-19 CT |  |  |  |  |  |  |  |  | - | .13** | .03 | .19*** | -.07 | .14** |
| 10. Justification of general violence |  |  |  |  |  |  |  |  |  | - | .63*** | .29*** | -.05 | .27*** |
| 11. Willingness for general violence |  |  |  |  |  |  |  |  |  |  | - | .26*** | -.06 | .19*** |
| 12. Paranoia |  |  |  |  |  |  |  |  |  |  |  | - | -.07 | .73*** |
| 13. Compliance with confinement |  |  |  |  |  |  |  |  |  |  |  |  | - | -.06 |
| 14. Vulnerable narcissism |  |  |  |  |  |  |  |  |  |  |  |  |  | - |
| *M*  (*SD*) | 4.43  (1.16) | 1.93  (1.38) | 2.08  (1.20) | 1.72  (1.22) | 1.23  (0.74) | 1.11  (0.41) | 1.04  (0.35) | 3.36  (2.04) | 2.14  (1.77) | 1.79  (1.11) | 2.03  (1.28) | 2.61  (1.03) | 6.61 (0.82) | 2.71 (1.17) |

^†^*p* < .06, **p* < .05, ***p* < .01, *p* < .001.

# Table S2. Total, direct, and indirect effects of 5G COVID-19 conspiracy beliefs predicting non-violent responses, mediated by anger

| Predictor | Criterion | Total effect | Direct effect | Indirect effect |
| --- | --- | --- | --- | --- |
| 5G COVID-19 CT | Justification of non-violent responses to 5G COVID-19 CT | **.22 [.09, .36]** | **.22 [.08, .35]** | .01 [-.01, .03] |
|  | Willingness for non-violent responses to 5G COVID-19 CT | **.42 [.31, .53]** | **.40 [.29, .51]** | **.02 [.001, .05]** |

*Note*. Significant effects are bolded for ease of viewing. CT = Conspiracy Theory. 95% bias-corrected confidence intervals used, along with 5000 bootstrap samples. Controlling for age, gender, education, and experience with COVID-19. Conspiracy mentality also used as a covariate.

# Table S3. Conspiracy beliefs predicting non-violent responses, mediated by anger, with the b-paths moderated by paranoia

| Criterion | Predictor | Coefficient | Index of moderated-mediation | Conditional indirect effects at levels of paranoia | | |
| --- | --- | --- | --- | --- | --- | --- |
|  |  |  |  | **Low** | **Moderate** | **High** |
| Justification of non-violent responses to 5G COVID-19 CT | | |  |  |  |  |
|  | 5G COVID-19 CT | **.22 [.09, .36]** |  |  |  |  |
|  | Anger | .04 [-.32, .40] |  |  |  |  |
|  | Paranoia | .23 [-.06, .52] |  |  |  |  |
|  | Anger x Paranoia | -.01 [-.12, .10] | -.002 [-.02, .01] | - | - | - |
| Willingness for non-violent responses to 5G COVID-19 CT | | |  |  |  |  |
|  | 5G COVID-19 CT | **.40 [.29, .51]** |  |  |  |  |
|  | Anger | .04 [-.25, .34] |  |  |  |  |
|  | Paranoia | .03 [-.21, .27] |  |  |  |  |
|  | Anger x Paranoia | .02 [-.07, .11] | .003 [-.007, .02] | - | - | - |

*Note*. Significant effects are bolded for ease of viewing. CT = Conspiracy Theory. 95% bias-corrected confidence intervals used, along with 5000 bootstrap samples. Controlling for age, gender, education, and experience with COVID-19. Conspiracy mentality also used as a covariate. Levels of the moderator are *M*–1*SD* (Low paranoia), *M* (Moderate paranoia), and *M*+1*SD* (High paranoia).

# Table S4. Total, direct, and indirect effects of conspiracy mentality predicting violent and non-violent responses to an alleged link between 5G and COVID-19, mediated by anger

| Criterion | Total effect | Direct effect | Indirect effect |
| --- | --- | --- | --- |
| Justification of real-life violent responses to 5G COVID-19 CT | .02 [-.06, .11] | .01 [-.08, .09] | **.01 [.0005, .04]** |
| Willingness for real-life violent responses to 5G COVID-19 CT | .008 [-.05, .06] | -.006 [-.06, .05] | **.01 [.003, .03]** |
| Justification of violent responses to 5G COVID-19 CT | .01 [-.02, .04] | -.003 [-.03, .03] | **.01 [.004, .03]** |
| Willingness for violent responses to 5G COVID-19 CT | -.01 [-.04, .02] | -.02 [-.05, .01] | **.01 [.001, .02]** |
| Justification of non-violent responses to 5G COVID-19 CT | **.47 [.32, .63]** | **.46 [.31, .62]** | .01 [-.01, .04] |
| Willingness for non-violent responses to 5G COVID-19 CT | **.32 [.20, .45]** | **.30 [.17, .43]** | **.02 [.003, .06]** |

*Note*. Significant effects are bolded for ease of viewing. CT = Conspiracy Theory. 95% bias-corrected confidence intervals used, along with 5000 bootstrap samples. Controlling for 5G COVID-19 conspiracy beliefs, age, gender, education, and experience with COVID-19.

# Table S5. Conspiracy mentality predicting violent and non-violent responses to an alleged link between 5G and COVID-19, mediated by anger, with the b-paths moderated at three levels of paranoia

| Criterion | Predictor | Coefficient | Index of moderated-mediation | Conditional indirect effects at levels of paranoia | | |
| --- | --- | --- | --- | --- | --- | --- |
|  |  |  |  | **Low** | **Moderate** | **High** |
| Justification of real-life violent responses to 5G COVID-19 CT | | |  |  |  |  |
|  | Conspiracy mentality | -.003 [-.09, .08] |  |  |  |  |
|  | Anger | -.10 [-.30, .10] |  |  |  |  |
|  | Paranoia | -.01 [-.17, .15] |  |  |  |  |
|  | Anger x Paranoia | .05 [-.01, .11] | ***.01 [-.0002, .02]*** | -.004 [-.02, .02] | .005 [-.009, .02] | ***.015 [-.0001, .04]*** |
| Willingness for real-life violent responses to 5G COVID-19 CT | | |  |  |  |  |
|  | Conspiracy mentality | -.006 [-.06, .05] |  |  |  |  |
|  | Anger | -.09 [-.22, .03] |  |  |  |  |
|  | Paranoia | -.09 [-.20, .01] |  |  |  |  |
|  | Anger x Paranoia | **.06 [.02, .10]** | **.01 [.001, .03]** | -.001 [-.02, .02] | .009 [-.002, .03] | .**020 [.005, .04]** |
| Justification of violent responses to 5G COVID-19 CT | | | |  |  |  |
|  | Conspiracy mentality | -.002 [-.03, .03] |  |  |  |  |
|  | Anger | **-.10 [-.17, -.03]** |  |  |  |  |
|  | Paranoia | **-.09 [-.17, -.03]** |  |  |  |  |
|  | Anger x Paranoia | **.05 [.03, .08]** | .**01 [.003, .02]** | -.002 [-.01, .01] | **.008 [.0004, .02]** | **.017 [.007, .04]** |
| Willingness for violent responses to 5G COVID-19 CT | | | |  |  |  |
|  | Conspiracy mentality | -.02 [-.04, .01] |  |  |  |  |
|  | Anger | -.06 [-.12, .005] |  |  |  |  |
|  | Paranoia | -.06 [-.12, -.01] |  |  |  |  |
|  | Anger x Paranoia | **.03 [.01, .05]** | ***.006 [-.0002, .02]*** | -.001 [-.01, .02] | .005 [-.001, .21] | **.011 [.003, .03]** |
| Justification of non-violent responses to 5G COVID-19 CT | | |  |  |  |  |
|  | Conspiracy mentality | .**43 [.27, .59]** |  |  |  |  |
|  | Anger | .04 [-.32, .40] |  |  |  |  |
|  | Paranoia | .23 [-.06, .52] |  |  |  |  |
|  | Anger x Paranoia | -.01 [-.12, .10] | -.003 [-.02, .02] | - | - | - |
| Willingness for non-violent responses to 5G COVID-19 CT | | |  |  |  |  |
|  | Conspiracy mentality | .**29 [.16, .42]** |  |  |  |  |
|  | Anger | .04 [-.25, .34] |  |  |  |  |
|  | Paranoia | .03 [-.21, .27] |  |  |  |  |
|  | Anger x Paranoia | .02 [-.07, .11] | .004 [-.01, .02] | - | - | - |

*Note*. Significant effects are bolded and marginal effects are bolded and italicised for ease of viewing. CT = Conspiracy Theory. 95% bias-corrected confidence intervals used, along with 5000 bootstrap samples. Controlling for 5G COVID-19 conspiracy beliefs, age, gender, education, and experience with COVID-19.

# Table S6. 5G COVID-19 conspiracy beliefs predicting violent and non-violent responses, mediated by anger, with the b-paths moderated at three levels of narcissism

| Criterion | Predictor | Coefficient | Index of moderated-mediation | Conditional indirect effects at levels of narcissism | | |
| --- | --- | --- | --- | --- | --- | --- |
|  |  |  |  | **Low** | **Moderate** | **High** |
| Justification of real-life violent responses to 5G COVID-19 CT | | |  |  |  |  |
|  | 5G COVID-19 CT | .**43 [.36, .51]** |  |  |  |  |
|  | Anger | .05 [-.14, .23] |  |  |  |  |
|  | Narcissism | -.03 [-.17, .12] |  |  |  |  |
|  | Anger x Narcissism | .01 [-.05, .07] | .001 [-.01, .01] |  |  |  |
| Willingness for real-life violent responses to 5G COVID-19 CT | | |  |  |  |  |
|  | 5G COVID-19 CT | .**18 [.13, .23]** |  |  |  |  |
|  | Anger | .03 [-.09, .15] |  |  |  |  |
|  | Narcissism | -.03 [-.12, .06] |  |  |  |  |
|  | Anger x Narcissism | .02 [-.02, .05] | .002 [-.005, .01] |  |  |  |
| Justification of violent responses to 5G COVID-19 CT | | | |  |  |  |
|  | 5G COVID-19 CT | **-.11 [-.16, -.06]** |  |  |  |  |
|  | Anger | **-.13 [-.19, -.06]** |  |  |  |  |
|  | Narcissism | **-.11 [-.16, -.06]** |  |  |  |  |
|  | Anger x Narcissism | **.06 [.04, .08]** | **.007 [.001, .02]** | -.004 [-.018, .002] | **.005 [.0002, .012]** | **.01 [.002, .04]** |
| Willingness for violent responses to 5G COVID-19 CT | | | |  |  |  |
|  | 5G COVID-19 CT | .**06 [.04, .08]** |  |  |  |  |
|  | Anger | .01 [-.05, .07] |  |  |  |  |
|  | Narcissism | -.03 [-.07, .02] |  |  |  |  |
|  | Anger x Narcissism | .01 [-.01, .03] | .001 [-.002, .007] |  |  |  |
| Justification of non-violent responses to 5G COVID-19 CT | | |  |  |  |  |
|  | 5G COVID-19 CT | **.22 [.09, .36]** |  |  |  |  |
|  | Anger | -.05 [-.39, .29] |  |  |  |  |
|  | Narcissism | .06 [-.19, .32] |  |  |  |  |
|  | Anger x Narcissism | .02 [-.08, .12] | .003 [-.009, .02] |  |  |  |
| Willingness for non-violent responses to 5G COVID-19 CT | | |  |  |  |  |
|  | 5G COVID-19 CT | **.40 [.29, .51]** |  |  |  |  |
|  | Anger | .07 [-.21, .34] |  |  |  |  |
|  | Narcissism | -.05 [-.26, .17] |  |  |  |  |
|  | Anger x Narcissism | .02 [-.06, .11] | .003 [-.007, .02] |  |  |  |

*Note*. Significant effects are bolded for ease of viewing. CT = Conspiracy Theory. 95% bias-corrected confidence intervals used, along with 5000 bootstrap samples. Controlling for conspiracy mentality, age, gender, education, and experience with COVID-19.

# Table S7. Conspiracy mentality predicting violent and non-violent responses, mediated by anger, with the b-paths moderated at three levels of narcissism

| Criterion | Predictor | Coefficient | Index of moderated-mediation | Conditional indirect effects at levels of narcissism | | |
| --- | --- | --- | --- | --- | --- | --- |
|  |  |  |  | **Low** | **Moderate** | **High** |
| Justification of real-life violent responses to 5G COVID-19 CT | | |  |  |  |  |
|  | Conspiracy mentality | .01 [-.08, .10] |  |  |  |  |
|  | Anger | .05 [-.14, .23] |  |  |  |  |
|  | Narcissism | -.03 [-.17, .12] |  |  |  |  |
|  | Anger x Narcissism | .01 [-.05, .07] | .002 [-.01, .02] |  |  |  |
| Willingness for real-life violent responses to 5G COVID-19 CT | | |  |  |  |  |
|  | Conspiracy mentality | -.006 [-.06, .05] |  |  |  |  |
|  | Anger | .03 [-.09, .15] |  |  |  |  |
|  | Narcissism | -.03 [-.12, .06] |  |  |  |  |
|  | Anger x Narcissism | .02 [-.02, .05] | .003 [-.01, .01] |  |  |  |
| Justification of violent responses to 5G COVID-19 CT | | | |  |  |  |
|  | Conspiracy mentality | -.002 [-.03, .03] |  |  |  |  |
|  | Anger | **-.13 [-.19, -.06]** |  |  |  |  |
|  | Narcissism | **-.11 [-.16, -.06]** |  |  |  |  |
|  | Anger x Narcissism | **.06 [.04, .08]** | **.01 [.003, .03]** | -.006 [-.02, .004] | **.007 [ .002, .02]** | **.02 [.01, .04]** |
| Willingness for violent responses to 5G COVID-19 CT | | | |  |  |  |
|  | Conspiracy mentality | .02 [-.006, .04] |  |  |  |  |
|  | Anger | .02 [-.05, .08] |  |  |  |  |
|  | Narcissism | -.03 [-.08, .02] |  |  |  |  |
|  | Anger x Narcissism | .01 [-.01, .03] | .003 [-.005, .01] |  |  |  |
| Justification of non-violent responses to 5G COVID-19 CT | | |  |  |  |  |
|  | Conspiracy mentality | **.45 [.29, .61]** |  |  |  |  |
|  | Anger | -.05 [-.39, .29] |  |  |  |  |
|  | Narcissism | .06 [-.19, .32] |  |  |  |  |
|  | Anger x Narcissism | .02 [-.08, .12] | .004 [-.02, .03] |  |  |  |
| Willingness for non-violent responses to 5G COVID-19 CT | | |  |  |  |  |
|  | Conspiracy mentality | **.30 [.17, .43]** |  |  |  |  |
|  | Anger | .07 [-.21, .34] |  |  |  |  |
|  | Narcissism | -.05 [-.26, .17] |  |  |  |  |
|  | Anger x Narcissism | .02 [-.06, .11] | .004 [-.01, .03] |  |  |  |
| Justification of general violence | | |  |  |  |  |
|  | Conspiracy mentality | **.15 [.08, .22]** |  |  |  |  |
|  | Anger | -.01 [-.19, .17] |  |  |  |  |
|  | Narcissism | .03 [-.10, .17] |  |  |  |  |
|  | Anger x Narcissism | .03 [-.02, .08] | .007 [-.006, .02] |  |  |  |
| Willingness for general violence | | |  |  |  |  |
|  | Conspiracy mentality | **.17 [.09, .26]** |  |  |  |  |
|  | Anger | .05 [-.16, .26] |  |  |  |  |
|  | Narcissism | .06 [-.10, .22] |  |  |  |  |
|  | Anger x Narcissism | .003 [-.06, .07] | .001 [-.01, .02] |  |  |  |

*Note*. Significant effects are bolded for ease of viewing. CT = Conspiracy Theory. 95% bias-corrected confidence intervals used, along with 5000 bootstrap samples. Controlling for 5G COVID-19 conspiracy beliefs, age, gender, education, and experience with COVID-19.

# Table S8. Total, direct, and indirect effects of conspiracy beliefs predicting compliance to confinement instructions, mediated by anger

| Predictor | Criterion | Total effect | Direct effect | Indirect effect |
| --- | --- | --- | --- | --- |
| 5G COVID-19 CT | Compliance to confinement | -.08 [-.14, -.02] | -.08 [-.14, -.02] | -.001 [-.01, .005] |
| Conspiracy mentality | Compliance to confinement | .008 [-.06, .07] | .01 [-.06, .08] | -.002 [-.01, .01] |

*Note*. CT = Conspiracy Theory. 95% bias-corrected confidence intervals used, along with 5000 bootstrap samples. Controlling for 5G COVID-19 conspiracy beliefs, age, gender, education, and experience with COVID-19.

# Table S9. Conspiracy beliefs predicting compliance to confinement, mediated by anger, with the b-paths moderated at three levels of the moderator

| Predictor | | Coefficient | | | | Index of moderated-mediation | | | Conditional indirect effects at levels of moderator  (either paranoia or narcissism) | | | |
| --- | --- | --- | --- | --- | --- | --- | --- | --- | --- | --- | --- | --- |
|  | |  | | | |  | | | **Low** | | **Moderate** | **High** |
| 5G COVID-19 CT | | **-.07 [-.13, -.02]** | | | |  | | |  | |  |  |
| Anger | | **.25 [.10, .41]** | | | |  | | |  | |  |  |
| Paranoia | | .14 [.02, .27] | | | |  | | |  | |  |  |
| Anger x Paranoia | | **-.08 [-.13, -.04]** | | | | **-.01 [-.03, -.001]** | | | **.014 [.001, .04]** | | .004 [-.001, .02] | -.006 [-.03, .001] |
|  | | |  | |  | | |  | |  |  |  |
| Conspiracy mentality | | .01 [-.06, .08] | | | |  | | |  | |  |  |
| Anger | | **.25 [.10, .41]** | | | |  | | |  | |  |  |
| Paranoia | | **.14 [.02, .27]** | | | |  | | |  | |  |  |
| Anger x Paranoia | | **-.08 [-.13, -.04]** | | | | **-.02 [-.04, -.004]** | | | **.021 [.006, .05]** | | .006 [-.002, .02] | -.010 [-.03, .003] |
|  |  | | |  | | |  |  |  |  |  |  |
| 5G COVID-19 CT | | **-.08 [-.13, -.02]** | | | |  | | |  | |  |  |
| Anger | | .12 [-.02, .27] | | | |  | | |  | |  |  |
| Narcissism | | .09 [-.02, .20] | | | |  | | |  | |  |  |
| Anger x Narcissism | | ***-.04 [-.09, .0001]*** | | | | **-.005 [-.02, -.0003]** | | | .006 [-.002, .02] | | .001 [-.007, .01] | -.005 [-.02, .001] |
|  |  | | |  | | |  |  |  |  |  |  |
| Conspiracy mentality | | .01 [-.06, .08] | | | |  | | |  | |  |  |
| Anger | | .12 [-.02, .27] | | | |  | | |  | |  |  |
| Narcissism | | .09 [-.02, .20] | | | |  | | |  | |  |  |
| Anger x Narcissism | | ***-.04 [-.09, .0001]*** | | | | **-.008 [-.02, -.001]** | | | .01 [-.003, .03] | | .001 [-.01, .01] | -.008 [-.03, .003] |

*Note*. Significant effects are bolded and marginal effects are bolded and italicised for ease of viewing. CT = Conspiracy Theory. 95% bias-corrected confidence intervals used, along with 5000 bootstrap samples. Controlling for age, gender, education, and experience with COVID-19. Conspiracy mentality also controlled for when 5G COVID-19 CT was predictor, and 5G COVID-19 CT controlled for when the conspiracy mentality was the predictor.

1. <https://aspredicted.org/blind.php?x=aq8qn7> [↑](#footnote-ref-1)
